# Violence against health care workers in China, 2013–2016: evidence from the national judgment documents

**DOI:** 10.1186/s12960-019-0440-y

**Published:** 2019-12-26

**Authors:** Ruilie Cai, Ji Tang, Chenhui Deng, Guofan Lv, Xiaohe Xu, Sean Sylvia, Jay Pan

**Affiliations:** 1 West China School of Public Health and West China Fourth Hospital, Sichuan University, No. 17, Section 3, Ren Min Nan Road, Chengdu, 610041 Sichuan China; 2 West China Research Center for Rural Health Development, Sichuan University, No. 17, Section 3, Ren Min Nan Road, Chengdu, 610041 Sichuan China; 3 School of Civil Aviation Security, Civil Aviation Flight University of China, NO. 46, Nanchang Road, Guanghan, 618307 Sichuan China; 4 School of Public Administration, Sichuan University, Chengdu, China; 5 Department of Sociology, University of Texas at San Antonio, One UTSA Circle, San Antonio, TX 78249 United States of America; 6 Gillings School of Global Public Health, University of North Carolina at Chapel Hill, 1101D McGavran-Greenberg Hall, CB#7411, Chapel Hill, NC 27599-7411 United States of America

## Abstract

**Background:**

Incidents of patient-initiated workplace violence against health care workers have been a subject of substantial public attention in China. Patient-initiated violence not only represents a risk of harm to health care providers but is also indicative of general tensions between doctors and patients which pose a challenge to improving health system access and quality. This study aims to provide a systematic, national-level characterization of serious workplace violence against health care workers in China.

**Methods:**

This study extracted data from the China Judgment Online System, a comprehensive database of judgment documents. Three key phrases, “criminal case,” “health care institution,” and “health care worker” were used to search the China Judgment Online System for relevant cases between January 1, 2013, and December 31, 2016. Data extracted from identified cases was used to document the occurrence, the degree of risk, and the factors associated with serious workplace violence.

**Results:**

In total, 459 criminal cases involving patient-initiated workplace violence against health care workers in China were reported and processed. The analysis revealed geographic heterogeneity in the occurrence of serious workplace violence, with lower incidence in western provinces compared to central and eastern provinces. Primary hospitals experienced the highest rates of serious workplace violence and emergency departments and doctors were at higher risk compared with other departments and health workers. Perpetrators were primarily male farmers aged 18 to 44 with low levels of education. The most frequently reported reasons of serious patient-initiated workplace violence included perceived medical malpractice by the perpetrator after the death of a patient, death of a patient with no other reason given, failures of the compensation negotiations after the death of a patient, and dissatisfaction with the treatment outcomes.

**Conclusions:**

Serious workplace violence against providers varies across regions and types of health care institutions in China. Perception of low-quality care is the most reported reason for violence. Efforts should be made to improve quality of care in the low-level health institutions and strengthen the doctor-patient communication during the whole course of service.

## Introduction

Health care workers are at high risk of being victims of patient-initiated workplace violence (WPV) around the world [1–10]. In the context of health care settings, WPV can be defined as incidents that health care workers or providers are abused, threatened, or assaulted in circumstances related to their work. It involves an explicit or implicit threat to health care workers’ safety, well-being, or health. Specifically, WPV can be physical or psychological, including but not limited to, verbal abuse, bullying, mobbing, pushing, biting, pinching, kicking, slapping, beating, stabbing, and/or shooting [11]. Consequences of WPV include a variety of adverse outcomes to health care workers as increased psychological stress, escalated staff turnover, diminished job satisfaction, decreased productivity, and reduced trust of management and co-workers [12–16]. Beyond consequences for health care workers themselves, WPV in health care settings may be indicative of general tensions between doctors and patients which can compromise health system access and quality [6, 17].

Data from small-scale surveys and media reports suggest that the incidence of WPV against health care workers in China has steadily increased over the past decade and become a serious, ubiquitous, and persistent social problem. A cross-sectional survey from 90 township hospitals of Heilongjiang province in 2014 found that 42.2% of health care workers had experienced WPV at least once in the past year, of which 8.8% had experienced both psychological and physical violence [18]. A survey from six hospitals located in Fujian province in 2014 reported that 48% of medical employees had experienced WPV in the past year [19]. And another survey conducted in two psychiatric hospitals which were from northern and southern China, respectively, showed that 82.4% of nurses had experienced at least one type of violent event in the past 6 months [20]. To synthesize these results, a meta-analysis reported that the overall prevalence of 44 related studies was 62.4% [21]. Recently, a national-level study identified 140 cases from 2010 to 2016 involving violence or disturbing public order because of medical malpractice. This study showed a significant relationship between violence and the death of a patient [22]. Events of fatal patient-initiated WPV were repeatedly and widely reported by the media [23]. For instance, from 2000 to 2011, 124 serious violence incidents in hospitals were publicized, including 29 murders and 52 serious injuries. Most of the reported victims were doctors [24].

However, existant research on this topic often mixed serious with non-serious WPV, which failed to distinguish the unique characteristics of the two different types of WPV. In addition, this body of research tends to be regional (e.g., provincial locales) or occupation-specific (e.g., among nurses or physicians), thus limited in scope [3, 25–29]. Given the widespread and negative impacts of serious WPV on the health care workforce and health care system, it is imperative to explore the contours of serious patient-initiated WPV in China. We aim to utilize a dataset extracted from a national-level database of court judgment documents to perform a comprehensive and descriptive analysis of serious patient-initiated WPV against health care workers in China.

## Methods

### Database

Data for this study were extracted from the judgment documents available in the China Judgment Online System (CJOS), which is operated and maintained by the Supreme People’s Court of China. The CJOS is an official website that posts the judgment documents containing all criminal cases processed and sentenced by any court in 31 provinces of the People’s Republic of China, with the exception of special cases concerning national security, juvenile delinquency, or inappropriate for publication on the internet by the People’s Court. The judgment documents have been routinely published within 7 days of sentencing since 2013. By the end of 2018, this online system contained more than 55 million judgment documents encompassing civil, administrative, and criminal cases.

### Search strategy and inclusion criteria

In this study, the severity of WPV against health care workers was determined by whether the violence perpetrator violated a criminal law judged by the court. All criminal judgment documents reported the date of crime and the date of sentence. While the former indicates the time when a crime occurred, the latter specifies the time when a judgment was rendered by the court. We extracted all the cases of serious patient-initiated WPV against health care workers occurring between January 1, 2013, and December 31, 2016. Data extraction and collection took place in March 2017. Since the CJOS was not established until 2013, January 1, 2013, was set as the study’s beginning date and December 31, 2016, was chosen as the study’s ending date.

Three key phrases were initially used to screen the judgment documents for serious patient-initiated WPV cases against health care workers. These phrases were “criminal cases,” “health care institution,” and “health care worker.” The phrase “criminal cases” was used in advanced search for the type of document, whereas the other two phrases served as the keywords in full text. The following more refined and specific terms, including “criminal cases,” “hospital,” “clinic,” “health center,” “health room,” “maternal and child health hospital,” “community health center,” “Centers for Disease Control and Prevention (CDC),” “physician,” “doctor,” “nurse,” “technician,” “pharmacist,” “laboratorian,” “medical personnel,” and “health worker,” were used to ascertain the cases potentially involving criminal cases of WPV against health care workers in health care settings. This initial search resulted in a total of 53 636 criminal cases.

**Fig. 1:**
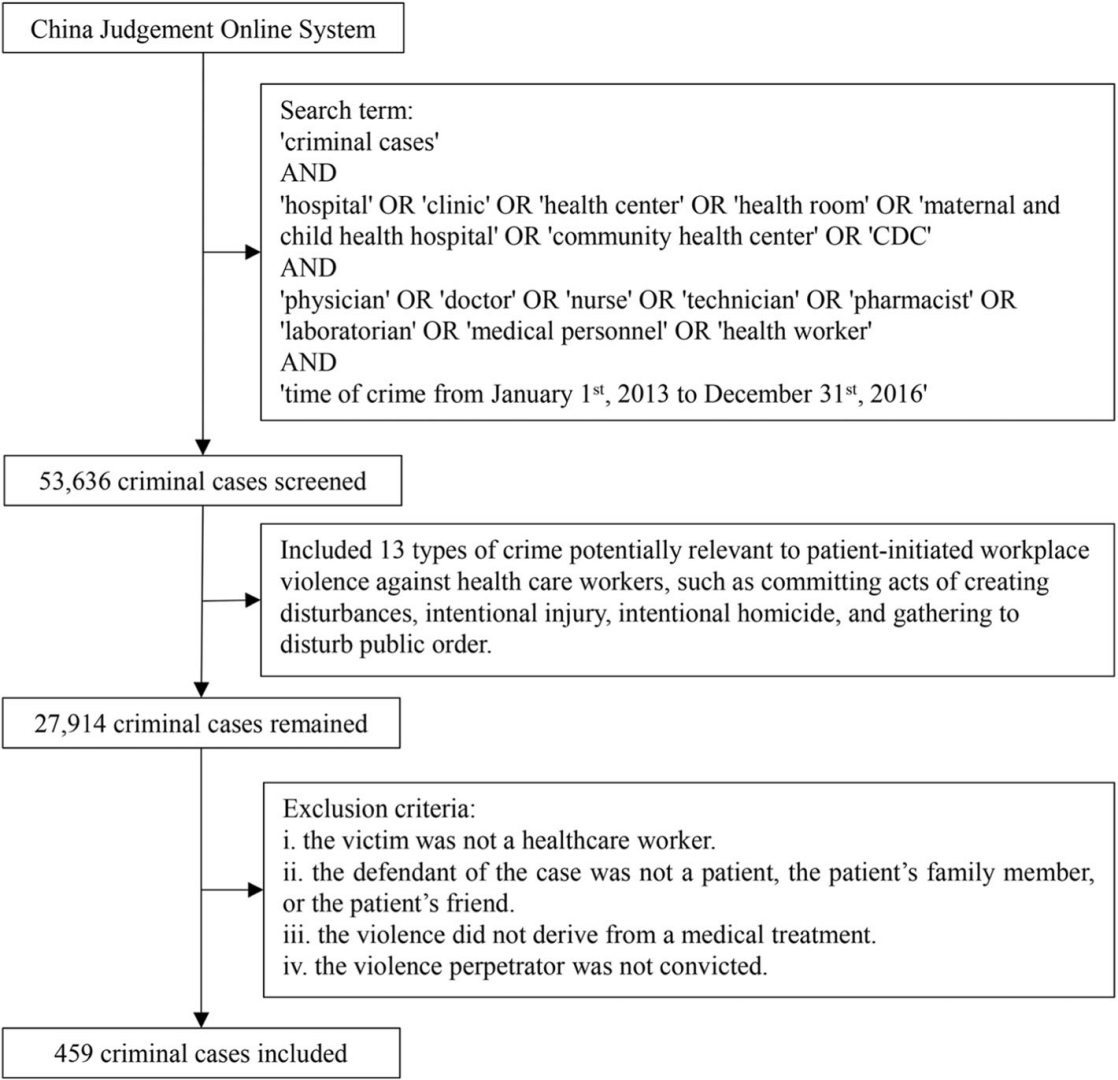
Flow chart of judgment document extraction

After all the judgment documents were extracted, they were carefully reviewed by four researchers separately to identify relevant cases. We categorized all the types of crime from the original 53 636 cases, which totally contained 88 crime types. Then, all crime types were screened according to the conviction conditions and applicable situations in consultation of jurisprudence expert on the research team. If a type of crime would possibly involve WPV towards health care workers, it would be included; otherwise, it was excluded. As a result, 13 types of crime were selected with a total of 27 914 potential cases, such as criminal acts of intentional injury, intentional homicide, and disturbing public order. Our study did not prescribe a first-sentence or second-sentence limit, but if both first instance and second instance of one violence case were found, we kept the second one to avoid the duplication. The exclusion criteria were (1) the victim was not a health care worker; (2) the violence perpetrator was not a patient, the patient’s friend, or the patient’s family member; (3) the violence was not related to medical care received; and (4) the violence perpetrator was not convicted. By enlisting these criteria, 459 non-duplicated cases were included in the present study, with 680 violence perpetrators in total. Figure 1 features the flow chart that details the document extracting process.

### Variable coding and statistical analysis

To prepare for the quantitative analysis, the extracted judgment documents were translated and coded into four groups of numerical and string variables in the standard data format. The first group of variables featured basic information about the criminal cases, including document number, court level, and the date of crime. The second group of variables gauged the characteristics of the health care workers involved in the WPV cases, including the type of health care worker, the type of health care institution, the location of health care institution, the level of hospital, and the type of department (e.g., the emergency department). The third group of variables reflected the characteristics of the perpetrator, including the name, age, gender, educational level, ethnicity, occupation, mental health conditions, and the category of violence initiated. The final group of variables contained the primary reported reasons for committing serious WPV against health care workers. The original data entry was performed by two individuals at the same time to establish inter-rater reliability. To account for potential variation across coders, the variable codes were carefully reviewed by four researchers independently. Discrepancies in variable coding were adjusted on the basis of the team’s consensus. Descriptive statistical analysis and hypothesis testing were conducted using R 3.3.1. Missing data were omitted when calculating the proportions, and the test level is set to 0.05.

## Results

### Regional variations

**Fig. 2:**
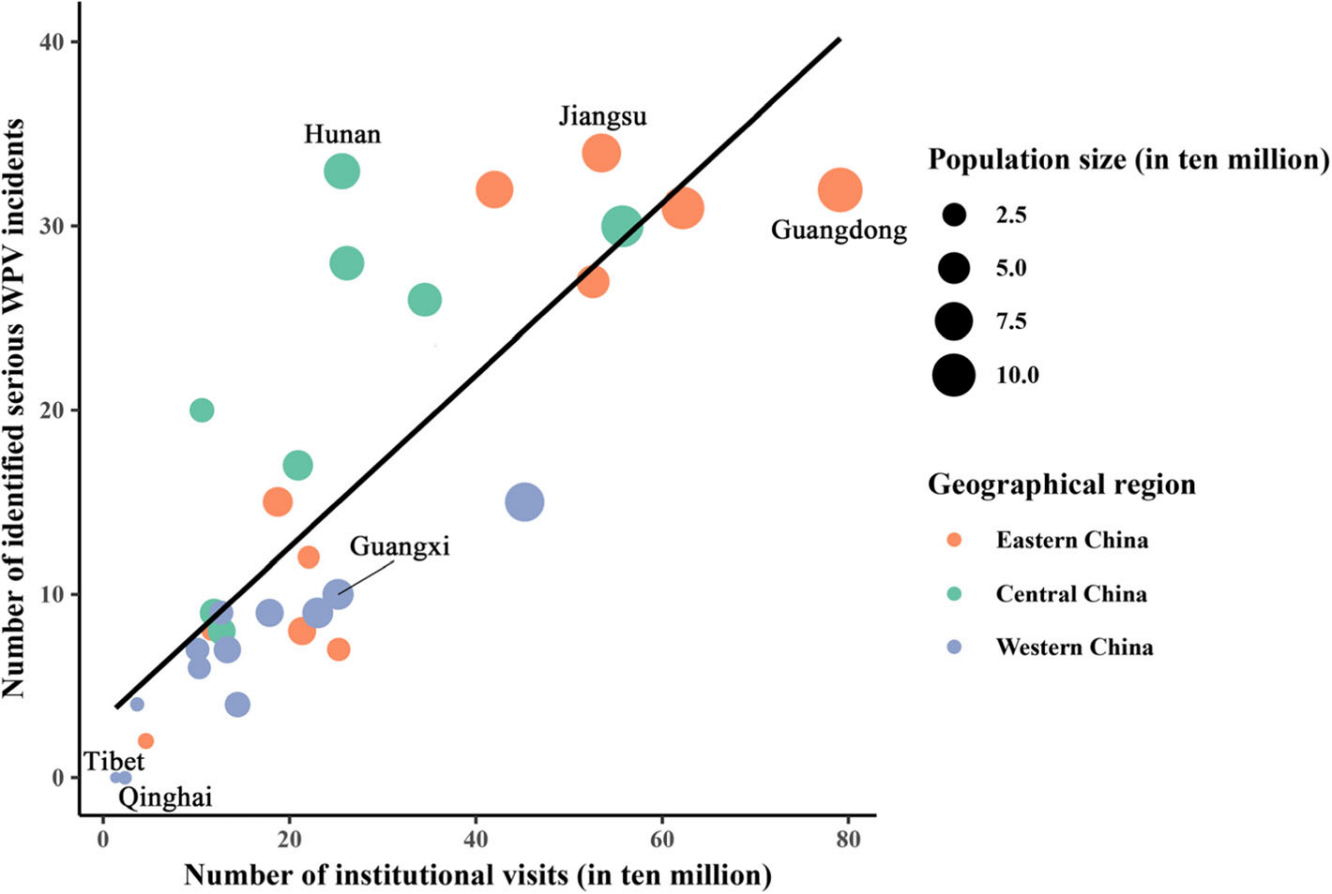
Serious patient-initiated workplace violence by region

Figure 2 plots variation in the distribution of the serious WPV cases against health care workers occurring between January 1, 2013, and December 31, 2016, by province. In the figure, the vertical axis represents the number of identified serious WPV incidents and each color-coded dot represents a province. Provinces were grouped into three broad geographical regions: western (blue), central (green), and eastern (orange). The level of socioeconomic development tends to increase from west to east in China. As shown in the figure, the majority of serious WPV cases occurred in the eastern region. While the highest number of cases were found in Jiangsu (*n* = 34; 7.40%) and Hunan province (*n* = 33; 7.19%), located in the eastern and central regions, respectively, no case was reported from Tibet (*n* = 0) or Qinghai province (*n* = 0), both of which are located in the west. The distribution of serious WPV against health care workers by province is displayed in Table 4 in the Appendix.

To account for the volume of institutional visits and population size, the horizontal axis in the figure indicates the total number of institutional visits and each dot represents population size in 10 million. These data plotted in the figure came from the 2014 to 2017 editions of China Health and Family Planning Statistics Yearbook [30–33]. As expected, the occurrence of serious WPV against health care workers was positively correlated with the number of institutional visits and population size. That is, as the number of institutional visits or population size increases, so did the number of identified serious WPV incidents.

The positive correlation between the number of cases and the number of institutional visits is further illustrated by the fitted line included in the figure. Vertical deviations from this fitted line illustrate the degree to which each province diverges from the expected number of cases on the basis of the linear relationship with the volume of hospital visits. A clear pattern emerged from the figure indicates that while all the provinces in the western region are below the fitted line, every province but one from the central region is above the fitted line, suggesting that the serious WPV incidence was lower in western China than in central China. For example, though Hunan and Guangxi provinces were similar in the number of institutional visits and in population size, the number of serious WPV occurrences in Hunan province was more than three times that of Guangxi province.

### Institutional variations

**Fig. 3:**
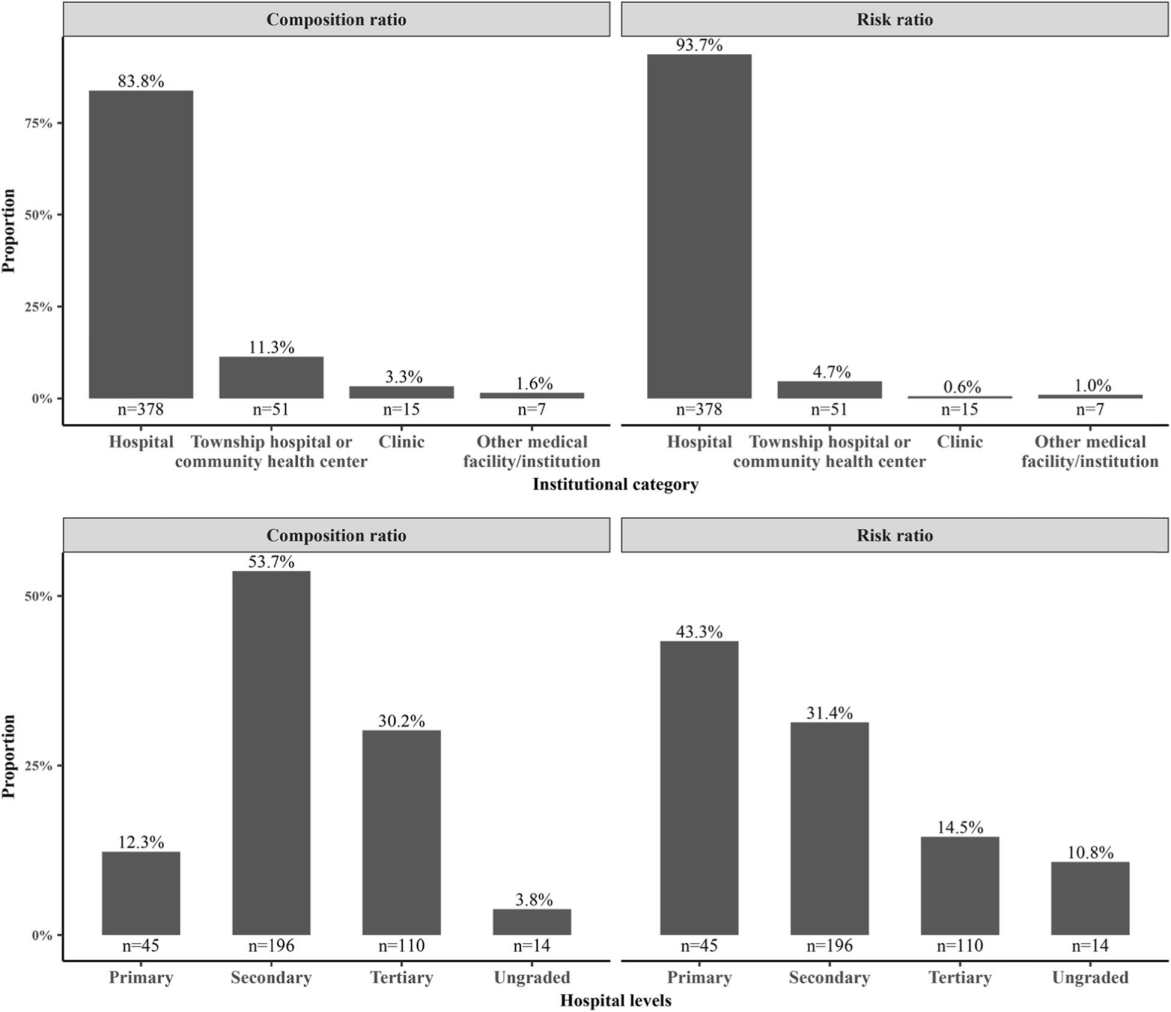
Occurrence and risk of serious patient-initiated workplace violence by health care institution. Note: eight cases were not reported for institutional category; 94 cases were not reported for hospital levels

Figure 3 shows the distribution of incidents by type of health care institution. Graphs on the left side of the figure display the composition ratio for each institutional category, calculated as the number of the serious WPV cases in each category divided by the sum of all cases. Graphs on the right side of the figure illustrate the risk ratio for each category, calculated as the composition ratio adjusted by the number of institutional visits in each category of health care institution between 2013 and 2016. Institutional variations can be demonstrated in both the type and level of each health care institution. The top panel of Fig. 3 shows distributions by type, categorized as city/county hospital, township hospital or community health center, clinic, or other medical facilities/institutions. As can be seen from the top panel, the vast majority of the serious WPV cases (83.8%) occurred in the city/county hospitals, which were also at the highest risk of experiencing serious patient-initiated WPV against health care workers (93.7%). The bottom panel of the figure reports institutional variations in the distribution of the serious WPV cases across levels of hospitals. Slightly more than half of the serious WPV cases (53.7%) occurred in the secondary hospitals. However, after adjusting for the number of hospital visits, the primary hospitals were about 1.4 times (43.3%) as likely as the secondary hospitals (31.3%) and three times as likely as the tertiary hospitals (14.5%) to experience serious patient-initiated WPV against health care workers.

### Departmental variations

**Fig. 4:**
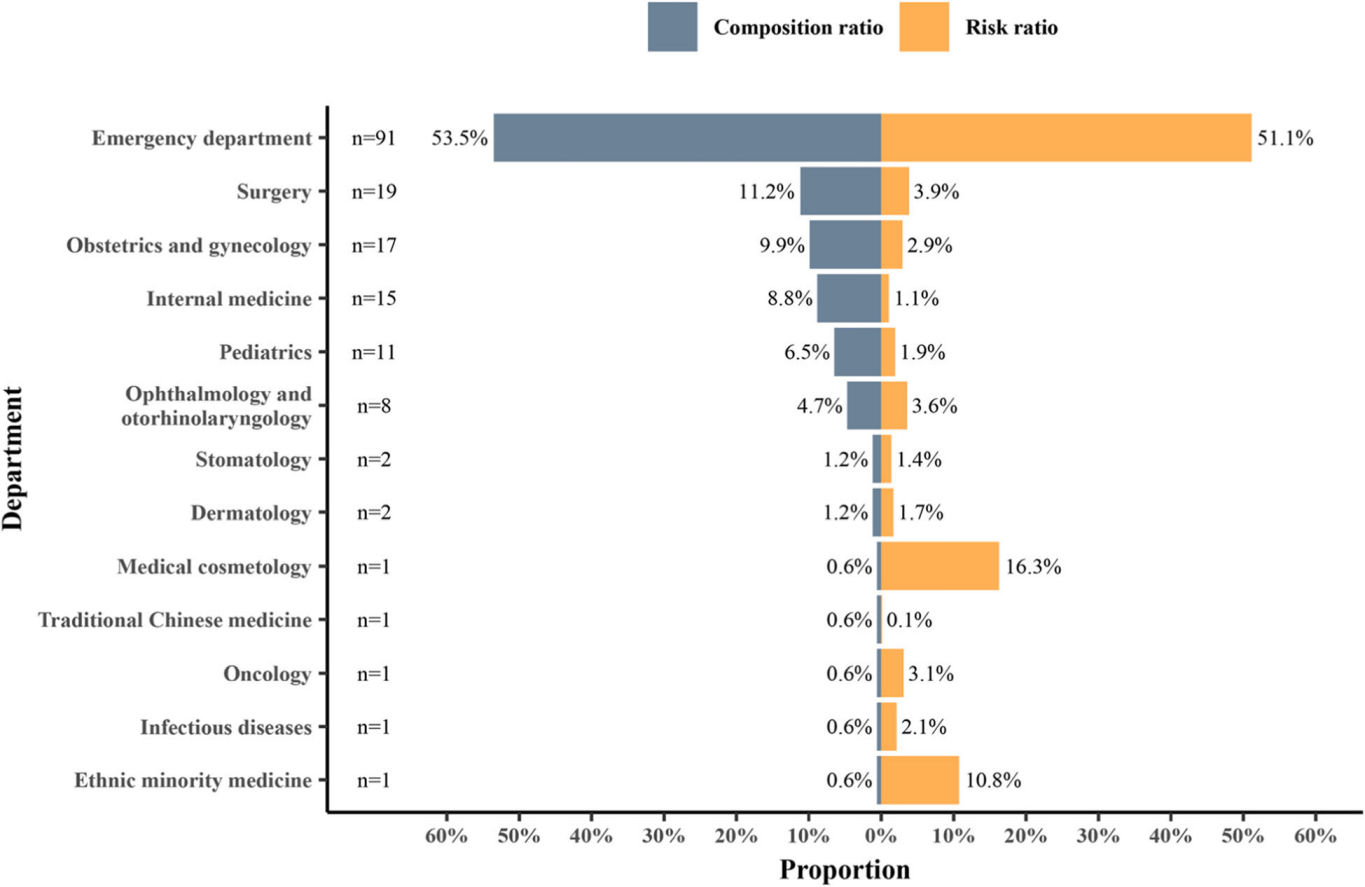
Occurrence and risk of serious patient-initiated workplace violence by department. Note: 289 cases were not reported

Using the same methods to compute the composition and risk ratios as displayed in Figs. 3 and 4 shows the intra-hospital distribution of cases across departments. Although cases were wildly distributed across 13 clinical departments, the emergency department accounted for the majority of the cases (53.5%), followed by the department of surgery (11.1%) and the department of obstetrics and gynecology (10.0%). After adjusting for the number of institutional visits, the emergency department was still at the highest risk of experiencing serious WPV against health care workers. Outside of the emergency department, however, the ranking of departments differed from the composition ratio in terms of risk. The department of surgery and the department of obstetrics and gynecology ranked second and third in terms of composition, while the medical cosmetology and the ethnic minority medicine were the second and third highest ranked departments in terms of risk.

### Occupational variations

**Fig. 5:**
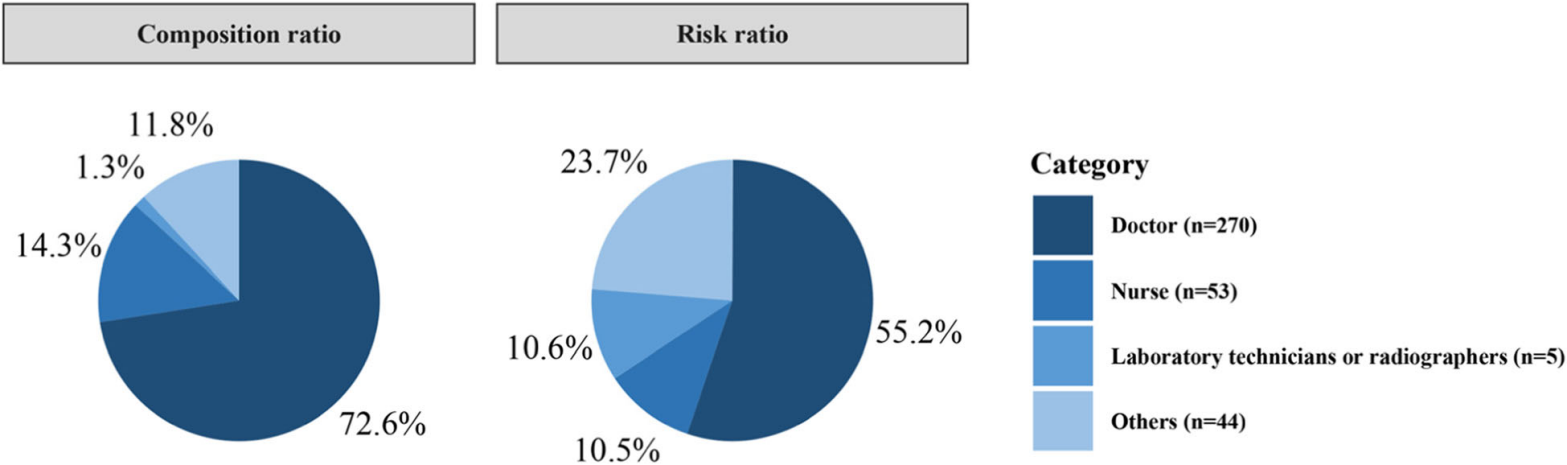
Occurrence and risk of serious patient-initiated workplace violence by victim’s occupation. Note: 87 cases were not reported

Although information about the victims (i.e., health care workers) of serious patient-initialed WPV in the judgment documents was limited, the type of occupation of health care workers was available. The occupational categories included doctor, nurse, laboratory technician or radiographer, and others (e.g., pharmacist, security office staff, and administrative personnel). Figure 5 exhibits the distribution of serious WPV cases across different victim occupations. The composition ratio is reported on the left side of the figure, and the risk ratio (adjusting for the number of workers in each occupation) is displayed on the right. The vast majority of the serious WPV cases were against doctors (72.6%), followed by nurses (14.3%). After adjusting for the number of health care workers in each occupation, the doctors, nurses, and laboratory technicians or radiographers were the top three occupations at risk of experiencing serious WPV. It should be noticed that although the composition ratio for laboratory technicians or radiographers was low (1.3%), their risk of experiencing serious WPV was relatively high (10.6%). The chi-square tests indicated that the rates of experiencing serious WPV are significantly different across occupations (*P* < 0.001).

### Characteristics of violence perpetrators

**Fig. 6:**
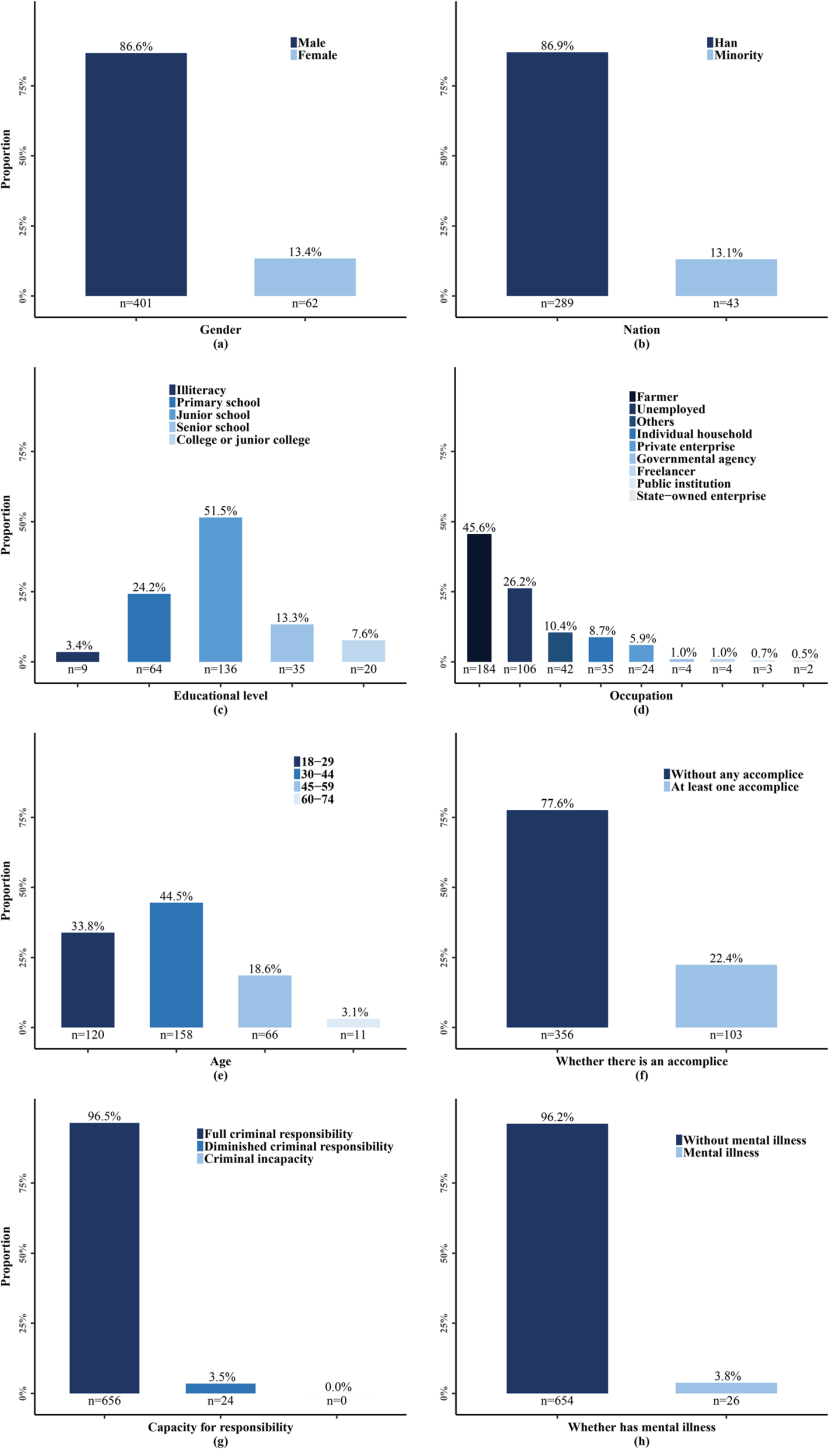
Characteristics of serious patient-initiated workplace violence perpetrators. Note: 217, 348, 416, 276, and 325 perpetrators were not reported from **a** to **e** while all the perpetrators were included in **f**, **g**, and **h**, respectively

Detailed information about the perpetrators of the serious WPV cases was included in the judgment documents. Figure 6 reports the perpetrators’ sociodemographic characteristics, including gender, age, education, ethnicity, occupation, mental illness, capacity for criminal responsibility, and accomplice status. The vast majority of the violence perpetrators were male (86.6%) and of Han ethnic origin (86.9%) and had little formal education (79.1% did not complete senior high school). Nearly half were farmers (45.6%) and 30–44 years of age (44.5%). The large majority of the violence perpetrators did not have an accomplice (77.6%) and bore full criminal responsibility (96.5%), and few were mentally ill according to the results of court-conducted psychiatric examinations (3.8%).

### Type of serious WPV and injuries

**Fig. 7:**
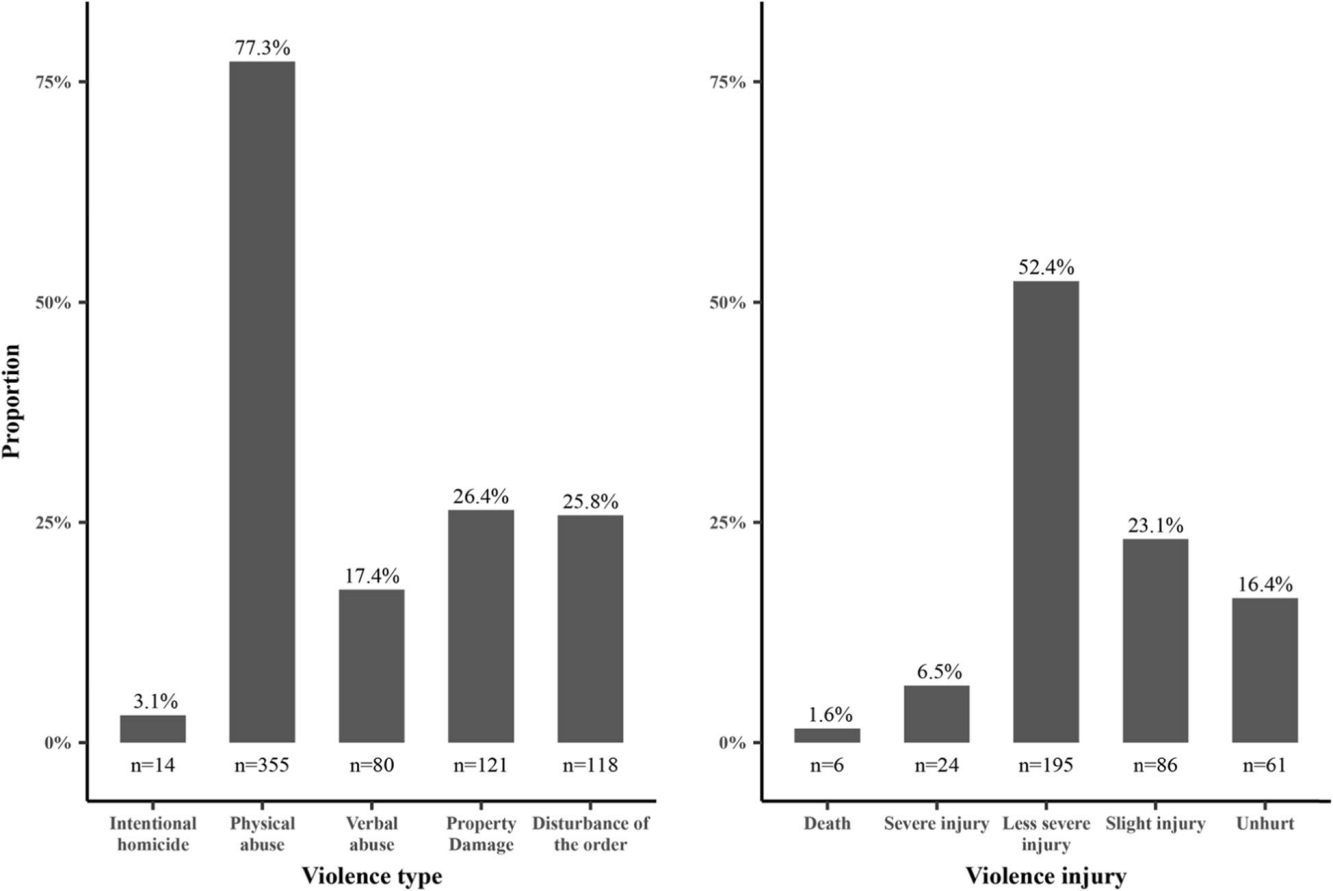
Type of serious patient-initiated workplace violence and injuries. Note: 87 cases were not reported for violence injury

Figure 7 displays the type of serious WPV against health care workers and the injuries inflicted. The violence was grouped into two broad categories, namely, violence against health care workers and violence against health care institutions. Serious WPV against health care workers included intentional homicide, physical abuse, and verbal abuse, whereas serious WPV against health care institutions encompassed property damage and disruption of public order. Since one incident might involve multiple types of violence, the sum across categories may exceed 100%.

As featured in the figure, more than half of the serious WPV cases involved both violence against health care workers and their institutions. The figure shows that 77.3% involved physical abuse, followed by property damage (26.4%), disruption of public order (25.7%), verbal abuse (17.4%), and intentional homicide (3.1%). The injuries inflicted were reported on the right side of the figure. All injuries were sorted in descending order by severity from the left to the right. According to the standard of appraisement of person’s injury degree [34], severe injuries included disability, disfigurement, auditory deprivation, and visual deprivation. Less severe injuries encompassed the appearance of damage and partial dysfunction with vision, hearing, or organs. Slight injuries referred to minor structural and/or functional damage to organs. More than half of the incidents (52.4%) resulted in less severe injuries, whereas 6.5% and 1.6% were severe injuries and deaths, respectively.

### Reported reasons for WPV against health care workers

Table 1 summarizes the major reported reasons for serious patient-initiated WPV against health care workers by treatment phase. Issues arising after treatment were the most frequently reported reasons for violence (35.34%), followed by those during treatment (22.51%). Issues prior to treatment were relatively few (8.38%). With the exception of the “other reasons” category (e.g., “trifles”), “death-related issues” (22.25%), “dissatisfaction with the treatment outcomes” (13.09%), and “dissatisfaction with the treatment process” (12.30%) were the top three reported reasons for serious WPV against health care workers. The category of death-related issues can be further decomposed into (1) “perceived medical malpractice by the perpetrator after the death of a patient” (8.38%), (2) “death of a patient with no other reason given” (7.59%), and (3) “failure of compensation negotiations after the death of a patient” (6.28%). It could be observed that the top three reasons, accounting for nearly half (47.64%) of all listed reasons, were relevant to the perceived quality of care.

**Table 1:** Reported reasons for serious patient-initiated WPV

Treatment phase	Reported reason	Proportion of incidents, *n* (%)
Before treatment	Long waiting time	20 (5.24)
	Rejection of request	12 (3.14)
During treatment	Dissatisfaction with the treatment process	47 (12.30)
	Dissatisfaction with staff attitude	39 (10.21)
After treatment	Death-related issues	85 (22.25)
	Dissatisfaction with the treatment outcome	50 (13.09)
Other reasons		89 (23.30)
No reason		40 (10.47)
Total		382 (100)

We further explored the associations between the reported reasons for serious WPV against health care workers and the victims’ occupation as well as the type of health care providers (i.e., hospital levels). The results are displayed in Tables 2 and 3. Considering only five laboratory technicians or radiographers are involved in serious WPV, we focus primarily on doctors and nurses that account for about 90% of all cases. Table 2 indicates that doctors are more likely to be victimized than nurses due to long waiting time (4.4% vs. 1.9%), rejection of request (2.2% vs. 1.9%), dissatisfaction with the treatment process (13.3% vs. 11.3%), death-related issues (8.9% vs. 1.9%), and dissatisfaction with the treatment outcome (13.0% vs. 5.7%). However, these differences are not statistically significant at the 0.05 level. It is also observed that more nurses than doctors were victimized by the perpetrators because of their dissatisfaction with their attitudes (18.9% vs. 7.4%). This difference is statistically significant (*P* = 0.021).

**Table 2:** Reported reasons for serious patient-initiated WPV by health care workers’ occupation

Treatment phase	Reported reason	Doctors (%)	Nurses (%)	*P*
Before treatment	Long waiting time	12 (4.4)	1 (1.9)	0.650^b^
	Rejection of request	6 (2.2)	1 (1.9)	1.000^b^
During treatment	Dissatisfaction with the treatment process	36 (13.3)	6 (11.3)	0.725^a^
	Dissatisfaction with staff attitude	20 (7.4)	10 (18.9)	0.021^a^
After treatment	Death-related issues	24 (8.9)	1 (1.9)	0.173^b^
	Dissatisfaction with the treatment outcome	35 (13.0)	3 (5.7)	0.170^a^
Other reasons		41 (15.2)	11 (20.8)	0.399^a^
No reason		24 (8.9)	12 (22.6)	0.012^a^
Total		198 (73.3)	45 (84.9)	

Note: The overall number of doctors and nurses in our study was set as the reference according to the literature (Li et al. [22]). A 2 × 2 table was formed by combining the overall numbers with numbers in each line and the hypothesis testing was conducted

^a^Pearson chi-square test

^b^The chi-square test with continuity correction

**Table 3:** Reported reasons for serious patient-initiated WPV by hospital levels

Treatment phase	Reported reason	Primary (%)	Secondary (%)	Tertiary (%)	*P*
Before treatment	Long waiting time	4 (8.9)	5 (2.6)	7 (6.4)	0.127^a^
	Rejection of request	3 (6.7)	3 (1.5)	3 (2.7)	0.144^b^
During treatment	Dissatisfaction with the treatment process	5 (11.1)	23 (11.7)	11 (10.0)	0.917^a^
	Dissatisfaction with staff attitude	7 (15.6)	14 (7.1)	10 (9.1)	0.274^a^
After treatment	Death-related issues	5 (11.1)	43 (21.9)	13 (11.8)	0.101^a^
	Dissatisfaction with the treatment outcome	8 (17.8)	16 (8.2)	10 (9.1)	0.217^a^
Other reasons		6 (13.3)	50 (25.5)	26 (23.6)	0.364^a^
No reason		2 (4.4)	23 (11.7)	10 (9.1)	0.378^a^
Total		40 (88.9)	177 (90.3)	90 (81.9)	

Note: The overall number of primary, secondary, and tertiary hospitals in our study was set as the reference according to the relevant research (Li et al. [22]). A 2 × 3 table was formed by combining the overall numbers with numbers in each line and the hypothesis testing was conducted

^a^Pearson chi-square test

^b^Fisher’s exact test

Turning to Table 3, it can be observed that compared to their secondary and tertiary counterparts, the primary hospitals are more likely to encounter WPV for such reasons as long waiting time (8.9% vs. 2.6%, 6.4%), rejection of request (6.7% vs. 1.5%, 2.7%), dissatisfaction with staff attitudes (15.6% vs. 7.1%, 9.1%), and dissatisfaction with the treatment outcome (17.8% vs. 8.2%, 9.1%). However, the secondary hospitals are more likely to encounter WPV than their primary and secondary counterparts due to death-related issues (21.9% vs. 11.1%, 11.8%). It should be noted that these differences are statistically negligible.

## Discussion

### Principal findings and interpretations

This study set out to develop and report a national profile of serious WPV against health care workers in China using data extracted from the judgment documents provided by the China Judgment Online System (CJOS), a publicly available online database. Through descriptive statistical analyses, several noteworthy results emerged, which will be summarized below.

This study revealed a geographical heterogeneity or regional variation in serious WPV against health care workers in China. It appeared that during the period of time from 2013 to 2016, serious WPV was more likely to occur in health care settings located in the eastern and central provinces than those located in the western provinces. Although the number of institutional visits and population size were identified as contributing factors to this regional variation, the health care system and policy factors, such as health regulation, health institutional management, health financing, and health care delivery settings, should be considered as potential contributing factors in future research endeavors. Moreover, judicial administration and crime rates at the provincial level should be taken into consideration as well in future research.

This study also found an institutional variation in the occurrence of serious WPV against health care workers in China. Generally, city/county hospitals faced higher levels of risk of experiencing serious WPV than other health care institutions. According to the interim measures for hospital evaluation [35], the primary hospitals demonstrated the highest risk of serious WPV after adjusting for the number of hospital visits, which was followed by the secondary and tertiary hospitals. While this finding was consistent with previous research [22], it is inconsistent with the media reports that often covered serious WPV in the tertiary hospitals. Results derived from this study suggest that policymakers should make greater efforts to prevent serious WPV in the primary hospitals that were often neglected in the past.

Disparities in the quality of health care provided by different levels of hospitals in China could be one of the key reasons to account for the institutional variations in serious WPV against health care workers. Higher-level hospitals in China typically hire better-qualified physicians and are often equipped with more advanced medical devices, which can in turn deliver a higher quality of care. On the other hand, lower-level hospitals tend to deliver a lower quality of care that can lead to seriously dissatisfied patients [36], thus resulting in more frequent serious WPV against health care workers. In recent years, the Chinese government has developed strategies for capacity building to strengthen low-level hospitals, which could help to reduce serious WPV against health care workers in these health institutions in the future [37].

The intra-institutional analysis results were consistent with the international studies [38, 39] in that the emergency department exhibits the highest incidence of serious WPV against health care workers in China. The emergency department routinely admits patients with the most complex and urgent conditions, including those who suffer from common diseases with acute complications and those who have acute diseases. Under such circumstances, patients as well as their relatives or friends may have a stronger tendency to express their discontent against health care workers. Our ancillary analysis of the judgment documents (not reported above) showed that 44% of the serious WPV cases occurred in the emergency department at night, while 18.5% of these cases reportedly involved alcohol. The impulsivity of drunken patients or their drunken companions can pose dangerous threats to health providers. Taken together, these results suggest that protective measures should be developed to prevent drunkenness-induced serious WPV in the emergency department, and the health workers must be vigilant about such threats.

Unexpectedly, however, our study indicated that the health care workers in the department of medical cosmetology were also at high risk of experiencing serious WPV against them. Since the 1990s, China has witnessed robust growth and high commercialization in medical cosmetology services for its profits. Many cosmetic departments of public hospitals have been transformed into private practices. However, due to the short-time expansion of medical cosmetology in China, the professional standards are under-developed and the regulatory supervision is inadequate. Medical cosmetology institutions routinely exaggerate their therapeutic effects and aggressively publicize their effectiveness in their advertisements [40]. Such commercial practices might have contributed to the growing frequency of medical disputes that could give rise to serious WPV.

Our study showed that doctors were at the highest risk of experiencing serious WPV in China. In fact, the risk of experiencing serious WPV for doctors was five times that of nurses. This finding, however, is at odds with previous studies that reported nurses having the highest risk of injury among health care workers [41–43]. Explanations for this apparent discrepancy are twofold. First, as explicated previously, our study focused primarily on serious WPV against doctors and nurses, whereas other studies included both serious and non-serious WPV. Second, even though doctors and nurses are both major health care providers in health care settings, doctors are the designers and practitioners of the medical diagnosis and treatment programs. Therefore, they are more likely to become targets of violence than nurses if the patients are dissatisfied with the quality of medical treatment, procedures, or outcomes. Hospitalized patients generally meet doctors once or twice a day, but they often receive nursing care several times a day. Therefore, infrequent communication between doctors and patients vis-à-vis nurses could be a potential contributing factor to the high frequency of serious WPV against doctors.

This study reported that the violence perpetrators were likely to be male, young or middle-aged, and poorly educated farmers. It is conceivable that patients or their family members with such characteristics in China would have low levels of medical literacy and unreasonable expectations of the medical procedures or treatment. As such, they might resort to violence if their expectations were not met. This can be particularly true for intoxicated perpetrators that seek medical care in the emergency department [44]. Given these characteristics associated with WPV perpetrators, developing medical literacy educational programs should be considered by policymakers.

A final major finding of this study was that most of the reported reasons for serious WPV against health care workers were dissatisfaction with treatment outcomes and/or deaths. Previous studies suggested that medical WPV often result from insufficient communication between hospital staff and patients, poor quality of medical treatment, dissatisfaction with treatment outcomes, and patients’ annoyance due to exorbitant medical/treatment cost [28]. The results reported in this study partially reflected the gap between the expected quality of care by patients and the service they actually received. The “dissatisfaction,” including the dissatisfaction with treatment process, staff attitudes, treatment outcomes, facilities, and cost, accounted for 32.9% of the reported reasons for serious WPV against health care workers. Given these results, improving the patient’s satisfaction with services and care could be one of the promising venues to reduce or prevent serious WPV against health care workers in China.

The disparity in reasons between violence against doctors and nurses, as well as different hospital levels, may provide policy implications to prevent WPV against health workers. Although statistically insignificant, serious WPV towards doctors occurred more before and after treatment, but less during the treatment. Moreover, the most frequently reported reasons in primary and secondary hospitals were long waiting time, rejection of request, dissatisfaction with staff attitude, dissatisfaction with the treatment outcome, and death-related issues. The patients seem to be more likely to impute the dissatisfied outcome (including a death) to doctors because the doctors were in charge of the treatments. The differences in reported reasons among different hospital levels may reflect lower quality of care in lower level hospitals.

### Strengths and limitations of study

This study has two major strengths. First, we used national database extracted from the China Judgment Online System, on which criminal judgment documents guarantee the authenticity and objectivity of our data. Second, compared with previous studies concerning WPV in China, our focusing on the serious WPV by clearly defining the severity of it would have more practical significance.

This study has several limitations. First, the descriptive statistical analysis conducted in this study may have overlooked potential confounding factors and complex interactions among these factors. As demonstrated previously, serious WPV against health care workers could arise from a host of factors, including regional, institutional, and individual correlates. Future research should explore these multifaceted and multilayered factors using experimental or quasi-experimental analyses. Second, this study may not include all the serious WPV cases. On the one hand, a small proportion of the criminal cases could take up to 1 year or more to be processed and prosecuted. As such, it is possible for the present study to miss the cases that occurred in the study period but yet to be available. On the other hand, some cases could be solved or mediated out of court, thus omitted from the present study. In addition, our research might have some selective bias if these cases have different distributions among regions, institutions, departments, and/or occupation types. As such, there might be discrepancies between the results reported here and the actual distributions of serious WPV against health care workers. Future studies are encouraged to collect these out-of-court cases to update this study and confirm the robustness of the results derived from the currently available database.

### Implications

Multifaceted approaches to preventing serious WPV against health care workers should be introduced to and implemented in all health care institutions, especially the primary and secondary hospitals as their high risk of experiencing serious WPV is likely to be neglected. Inasmuch as lack of medical literacy among patients contributes to dissatisfaction with the quality of care received, violence could be reduced through long-term public health literacy programs. Efforts to improve communication between providers and patients should be made, especially in the tertiary hospitals where patients’ dissatisfaction is more pronounced. Additionally, efforts to improve the quality of medical services should be made as well to reduce the incidence of undesirable treatment outcomes. Finally, it is recommended that the government regulations with regard to medical cosmetology be developed and reinforced.

Hospitals should prioritize the departments of emergency, surgery, obstetrics and gynecology, and internal medicine for violence prevention. Educational programs to train self-defense and aggression de-escalation techniques for individual health care workers should be developed [45]. Doctors and nurses in high-risk departments, especially in the emergency department, should raise awareness of WPV and impediments or barriers to report WPV incidents. More importantly, a pre-warning system of risk assessment could be devised in order to alert health care workers to the potential threats [15].

## Data Availability

The datasets generated and analyzed during the current study are available in the China Judgment Online System repository, http://wenshu.court.gov.cn/.

## Appendix

**Table 4:** Number of serious WPV among 31 provinces in China

Province	Number of identified serious WPV incidents	Geographical region
Beijing	12	Eastern China
Tianjin	8	Eastern China
Hebei	32	Eastern China
Shanxi	8	Central China
Inner Mongolia	7	Western China
Liaoning	15	Eastern China
Jilin	20	Central China
Heilongjiang	9	Central China
Shanghai	7	Eastern China
Jiangsu	34	Eastern China
Zhejiang	27	Eastern China
Anhui	28	Central China
Fujian	8	Eastern China
Jiangxi	17	Central China
Shandong	31	Eastern China
Henan	30	Central China
Hubei	26	Central China
Hunan	33	Central China
Guangdong	32	Eastern China
Guangxi	10	Western China
Hainan	2	Eastern China
Chongqing	4	Western China
Sichuan	15	Western China
Guizhou	7	Western China
Yunnan	9	Western China
Tibet	0	Western China
Shaanxi	9	Western China
Gansu	9	Western China
Qinghai	0	Western China
Ningxia	4	Western China
Xinjiang	6	Western China
Total	459	

## Abbreviations

CJOS: The China Judgment Online System
WPV: Workplace violence
